# Cooperation across multiple game theoretical paradigms is increased by fear more than anger in selfish individuals

**DOI:** 10.1038/s41598-021-88663-0

**Published:** 2021-04-30

**Authors:** G. Chierchia, F. H. Parianen Lesemann, D. Snower, T. Singer

**Affiliations:** 1 Department of Psychology, University of Cambridge, Cambridge, UK; 2 Utrecht University, Utrecht, The Netherlands; 3 Department of Economics, Hertie School of Governance, Berlin, Germany; 4 Blavatnik School of Government, Oxford, UK; 5 Social Neuroscience Lab, Max Planck Society, Berlin, Germany

**Keywords:** Psychology, Human behaviour

## Abstract

Cooperative decisions are well predicted by stable individual differences in social values but it remains unclear how they may be modulated by emotions such as fear and anger. Moving beyond specific decision paradigms, we used a suite of economic games and investigated how experimental inductions of fear or anger affect latent factors of decision making in individuals with selfish or prosocial value orientations. We found that, relative to experimentally induced anger, induced fear elicited higher scores on a cooperation factor, and that this effect was entirely driven by selfish participants. In fact, induced fear brought selfish individuals to cooperate similarly to prosocial individuals, possibly as a (selfish) mean to seek protection in others. These results suggest that two basic threat-related emotions, fear and anger, differentially affect a generalized form of cooperation and that this effect is buffered by prosocial value orientation.

## Introduction

Humans across cultures are known to frequently incur costs to benefit or punish others^1^. To account for this, psychologists^2,3^ have demonstrated that stable inter-individual differences in social value orientation (such as prosocial vs. individualistic value orientation) can influence such social decisions across time and contexts^4,5^. Similarly, economists have developed theories of social preferences, conceived as stable inter-individual differences in preferences for certain type of goals^6–8^. Much harder to reconcile with economic theory are ‘incidental’ or induced emotions and motives, which have been shown to affect decisions even if they are unrelated to them^9–11^. Here, we ask how individual differences in social value orientation modulate decisions to cooperate and punish when individuals feel threatened. We address this by assessing how experimental inductions of fear (threat-avoidance motives) and anger (threat-approach motives) affect these decisions in individuals with differing social value orientation.

A long-standing view contends that fear and anger arise from a common underlying threat system^12^, with fear promoting the avoidance of threats^13–15^, and anger their approach^16,17^. In line with this, both inter-individual differences in disposition to experience fear and anger^18–20^, as well as experimental inductions of fear and anger^21–26^, have been shown to oppositely affect judgment and decision making, with fear contributing to avoidance, and anger to approach of risk in non social decisions. However, it is unclear how this may extend to *social* decisions, such as those related to cooperation and punishment^27^.

In particular, while induced anger has been associated with decreased cooperation^28–30^ and increased punishments^31–35^, it remains unclear how fear may be related to cooperative decisions. On the one hand, cooperation frequently involves real or perceived “risks”, such as the risk of being socially excluded or the risk that one’s cooperation will not reciprocated. In this case, induced fear could lead individuals away from cooperation. On the other hand, fear has also been linked with a motive to seek protection and safety^13,15,36,37^, potentially leading subjects to engage in cooperative behaviors as a mean to seek protection in others^38^. Problematically, the empirical literature remains mixed in this regard, as manipulations of fear, or fear-associated stress, have been associated with both increased cooperative behaviors^39–44^ and decreased cooperative behaviors^45–47^.

One possible source for the inconsistency of these results could be that these studies focused on different decision paradigms, which varied in important characteristics. For instance, some studies focused on cooperative decisions that involved uncertainty of reciprocation (e.g., trusting others)^39,45,47^, while other studies did not (e.g., when deciding to give to others with no possibility of reciprocation)^40–42,45,46,48^. As briefly described above, even in non social decision making, induced fear can lead to aversion to risk and uncertainty^18^ and it is therefore unsurprising that this may carry over to social decisions in which uncertainty is salient^47^. The decision paradigms employed in previous studies also varied in other potentially important aspects: from specific payoff parameters^49^, to nuances of the instructions^50^, “details” that are now known to affect decisions^51,52^, and that could plausibly interact with the effects of induced emotions. These and many other differences make it difficult to compare existing results, and plausibly also reflect the more general problem that decision scenarios vary between laboratories, let alone in the field^53^.

One standard way to address this problem would be to “zoom into” each decision paradigm and systematically investigate the impact of induced emotions in each of its variants. However, this approach would be rather impractical as, for example, it would require a large amount of experiments and many corrections for multiple comparisons. Here, we take a more recent and contrasting approach that involves “zooming out” of specific decision paradigms, by focusing on sources of variance in cooperation and punishment that are common to many of them. In particular, recent studies have used factor analysis to individuate *latent constructs* of cooperation and punishment^54–56^. These and other studies suggest that despite critical differences between decision paradigms, subjects who tend to cooperate more in one decision paradigm, also tend to do so in others: from cooperation in social dilemmas (such as prisoner’s dilemmas) to unreciprocated giving (e.g., dictator games)^57^, to decisions to trust others, donating to charities, reciprocating kind gestures with kindness etc. Cooperative behavior has also been suggested to remain stable in time^4^, and to extend outside of laboratory settings^55^, leading some authors to speak of a “domain general […] cooperative phenotype”^55^ (p. 1), which has recently been extended to a “moral phenotype”^58^. Here, we thus aimed to investigate whether induced fear and anger may have such a generalized impact on decisions.

Finally, in addition to variation in decision paradigms, we aimed to control for variation between individuals. In particular, theories on attitude-behavior links suggest that strong attitudes towards a given behavior can make that behavior more stable in time, more resistant to change and more context independent^59,60^ and some findings indeed suggest that context effects on cooperation are greater in individuals with weak cooperative value orientations^61,62^. Moreover, with regards specifically to fear, other studies^44,48^ found that fear-related inductions tend to increase cooperative behaviors only in subjects with weak pre-existing attitudes towards those behaviors. Based on this, we hypothesized that strong prosocial value orientations could potentially buffer the influence of induced fear and anger on decisions. To measure such values we used the social value orientation task (henceforth, “SVO”)^2^, because in addition to being a well-known predictor of behavior in economic games^5,63^, it has also been shown to predict cooperative behavior across domains^4^, from volunteering^64^, to donating^65^, to engaging in pro-environmental efforts^66^.

In synthesis, while induced anger has been related to decreased cooperation and increased punishment, understanding how induced fear contributes to these behaviors has remained a contended issue, both theoretically and empirically. To assess generalizable results across decision paradigms and individuals, we aimed to compare the impact of induced fear and anger on latent factors of cooperation and punishment using a suite of game-theoretical paradigms and we proposed that the impact of such emotions should be primarily be observed in subjects that do not have already have strong cooperative value orientations to counteract them. These subjects could provide a good test-bed to clarify whether induced fear is more likely to increase or decrease cooperative behaviors.

## Results

### Induction validation

**Figure 1:**
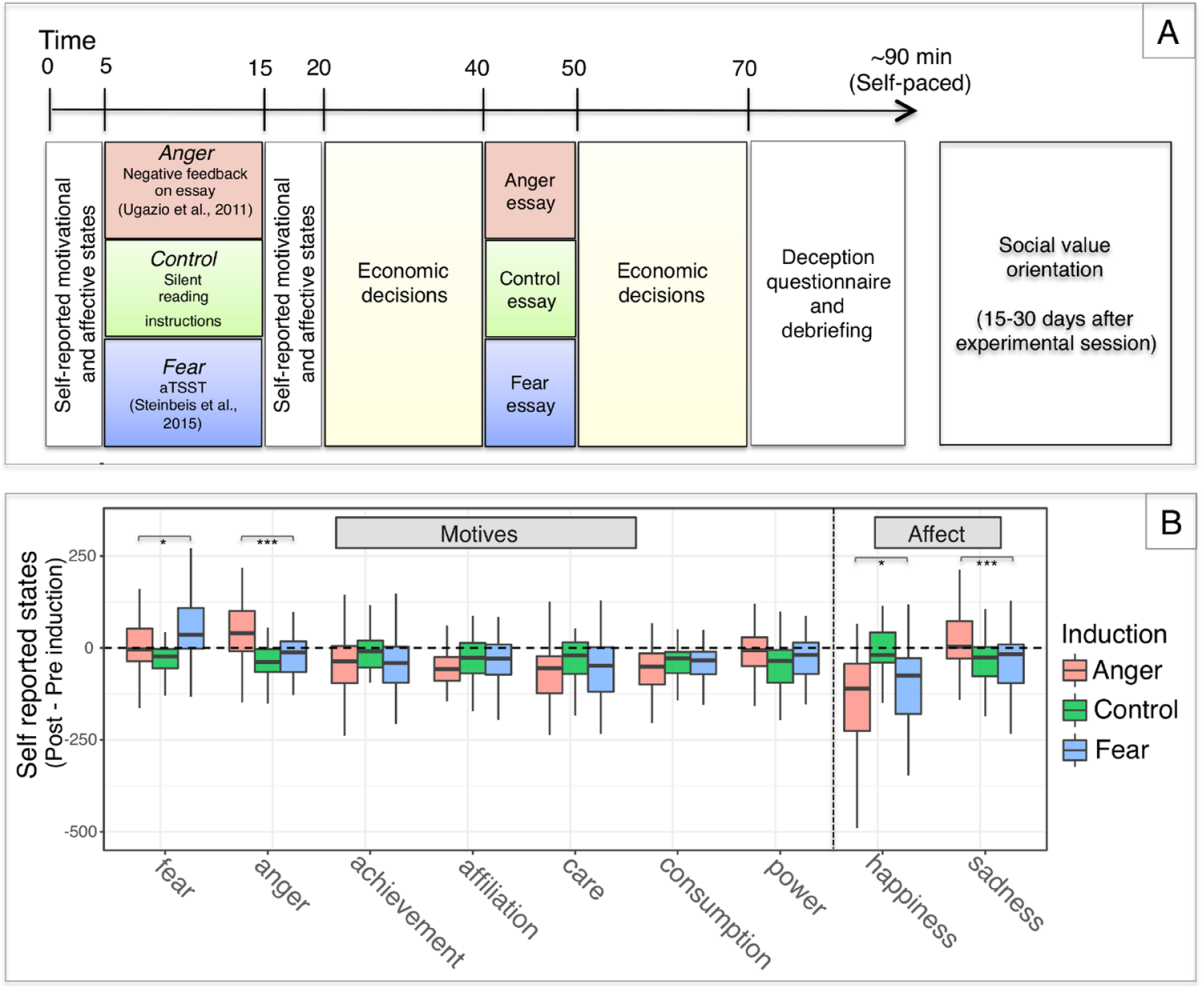
Panel (**A**) Experimental design. Panel (**B**) Induction validation. Planned contrasts showed that increases in anger ratings were higher in the Anger induction than in the Fear induction. Vice versa, increases in fear were higher in the Fear induction that in the Anger induction. Exploratory contrasts revealed that induced Fear and Anger also differentially elicited happiness and sadness. Ratings were provided on visual analogue scales ranging from − 350 to 350. ***p < 0.001, **p < 0.01, *p < 0.05, Bonferroni corrected.

To induce fear, one group of participants took part in an anticipatory version of the Trier Social Stress Task^45^, involving a simulated job interview. Another group of participants took part in an Anger induction (inductions will henceforth be capitalized to distinguish these from other occurrences of the emotion terms), in which they received negative and unfair feedback on a short essay they wrote^67^. In a Control induction, a third group of participants were requested to read a passage of text. These inductions were matched in terms of duration and sequence of events (Fig. 1A) (see “Inductions” in the Methods section for details). To validate these inductions, before and after being introduced to their respective activities, participants rated how well a number of words described their current mood, emotions and motives (see “Induction validation” in the Methods section for details). Inter-mixed amongst a number of control emotions and motives were words related to fear and anger. Changes in ratings were used as dependent variables to validate the inductions.

A linear mixed effect model on change scores (i.e., difference between post-induction and pre-induction ratings) revealed a significant interaction between the induction and the motive/affective state (F_(16,1376)_ = 15.007, p < 0.001), suggesting that the latter were differentially affected by the inductions (Fig. 1B). Contrasts within the model suggested that the Fear and Anger inductions succeeded in differentially eliciting the target motives: increases in anger were higher in the Anger induction than in the Fear induction (“Anger–Fear”: b = 91.192, 95% CI [30.768 96.324], p < 0.001) and in Control induction (“Anger–Control”: b = 91.192, 95% CI [59.338 123.047], p < 0.001); while increases in fear were higher in the Fear induction than in the Anger induction (“Anger–Fear”: b = − 39.222, 95% CI [− 74.998 − 9.442], p = 0.048) and in the Control induction (“Control–Fear”: b = − 91.976, 95% CI [− 123.831 − 60.122], p < 0.001) (p-values are Bonferroni-corrected for four tests). Exploratory contrasts on the other questionnaire items further showed that induced Fear and Anger did not elicit differential effects on other control motives, which included Power, Care, Affiliation, Achievement and an exploratory Consumption motive (p_s_ > 0.192, see Supplementary Material, Supplementary Table ST3 for all contrasts). However, relative to the Fear induction, the Anger induction resulted in increased sadness (“Anger–Fear”: b = 74.205, 95% CI [41.427 106.983], p < 0.001) and decreased happiness (“Anger–Fear”: b = − 53.007, 95% CI [− 85.785 − 20.229], p = 0.046). To investigate this further, we asked whether changes in anger and fear could be explained by changes in happiness and sadness. To do so, we used the anger and fear change scores as dependent variables, and ran two linear regression models on each. As independent variable, we used the induction factor and the changes in happiness or sadness ratings. These models suggested that, even though happiness and sadness significantly contributed to the increases in anger and fear (all p_s_ < 0.001, except for the impact of happiness on anger: p = 0.084), the inductions continued to reliably predict changes in the target motives (all p_s_ < 0.001), even controlling for the non-target changes in happiness and sadness. This suggests that changes in fear and anger were far from fully explained by changes in happiness and sadness. Nonetheless, in addition to these control models, we further controlled for these unanticipated effects of our inductions by adding happiness and sadness change scores as covariates when modeling the economic decisions (see “Model 2” in the Supplementary Material, Supplementary Tables ST4 and ST5).

### Two factors of economic behavior

While participants waited for the induction activities to take place, they took part in an allegedly separate study on economic decision making, involving real monetary incentives, and consisting in a suite of game theoretic paradigms (see “Game theoretic paradigms” in the Methods section for details). In contrast, social value orientation measures (SVO) were requested from participants 2 weeks after the inductions to avoid any spill-over effects between the two (see “Social value orientation” in the Methods section for details). Finally, an ‘experimental demand’ questionnaire was conducted after the game theoretic paradigms to probe participants’ awareness of any relation between these and the inductions (see Supplementary Material SM1, “Experimental demand questionnaire”).

**Figure 2:**
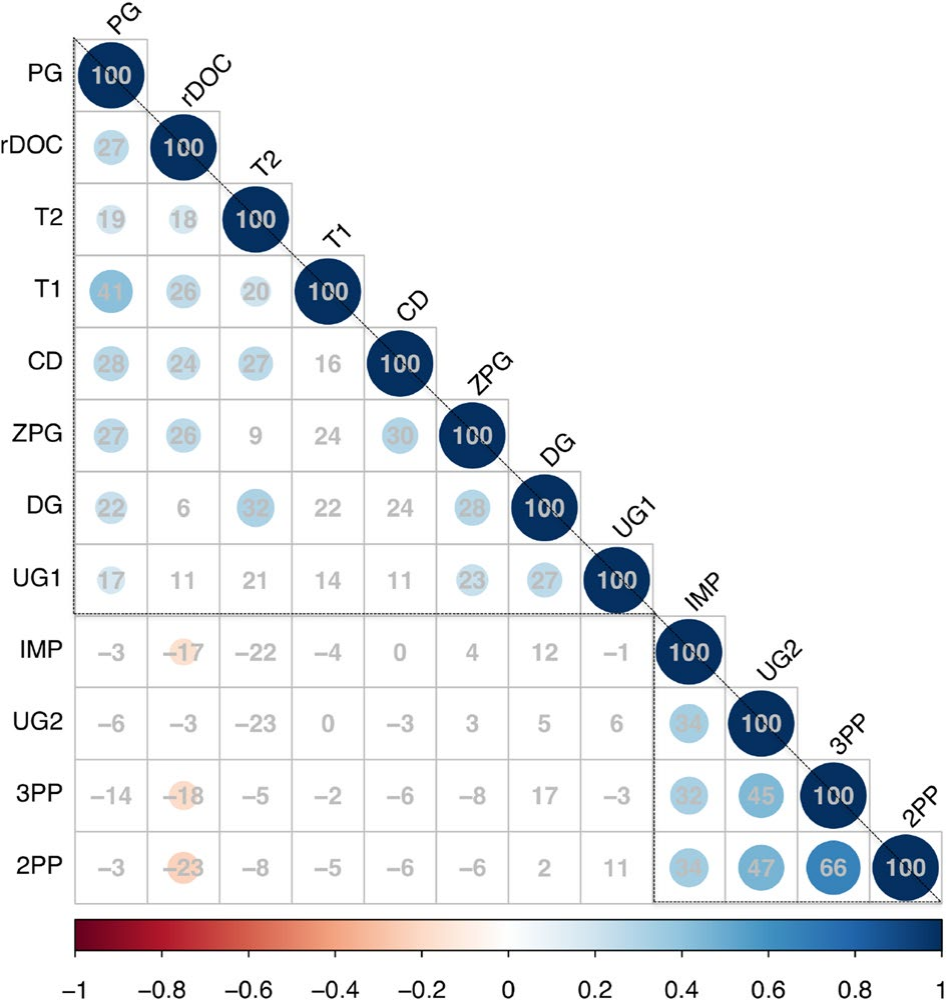
Spearman correlation matrix of behavior in game-theoretic paradigms. Colored cells represent significant correlations corrected with the Holm’s method. Numbers are correlation coefficients presented in percentages. Variables are ordered on the basis of the first principle component, triangles are manually super-imposed to highlight two potential groups of decisions. PG (contributions in a public good game), TG1 and TG2 (amounts entrusted and returned as 1st and 2nd mover in a trust game, respectively), rDOC (restraint in a dilemma of the commons), CD (amounts donated to charities), ZPG (frequency of helping choices in the Zurich prosocial game), DG (transfers in a dictator game), UG1 (offer size in an ultimatum game), UG2 (frequency of rejections in an ultimatum game), IMP (frequency of rejections in an impunity game), 2PP and 3PP (amount spent to punish norm violators in 2nd and 3rd party punishment games).

A Spearman’s correlation matrix (Fig. 2) suggested that two groups of economic behaviors were inter-correlated. Specifically, 1st and 2nd movers in the trust game—represented by average (1st mover) transfer rates (i.e., “trust” by 1st movers) and average returns (i.e., “trustworthiness” by 2nd movers), average charitable donations, transfers in the dictator game, proposals as 1st movers in the ultimatum game, contributions to public goods, restraint in common resource dilemmas and helping in the Zurich prosocial game, all positively correlated. On the other hand, average punishment rates in the 2nd and 3rd party punishment games, and frequency of rejections in the ultimatum and impunity games, all positively correlated with one another.

To formally test these groupings in a factor analysis, we first obtained a number of indexes indicating how many components to retain (see “Statistical analyses” in the Methods section for details). All tested indexes (parallel analysis, optimal coordinates, acceleration factor, very simple structure and Velicer’s MAP criterion) recommended retaining 2 factors. Moreover, since oblique (i.e., oblimin) rotation revealed only a very weak (anti-) correlation between the two factors (r = − 0.1), we retained Varimax rotation^68^. The resulting orthogonal 2-factor solution had a satisfactory fit (RMSEA = 0.064, TLI = 0.852) and confirmatory factor analysis suggested it was stable across the inductions: nor the loadings nor the intercepts differed between the inductions (respectively p = 0.122 and p = 0.659). It also explained nearly one third (31%) of the variance of 12 economic variables with only two factors.

**Figure 3:**
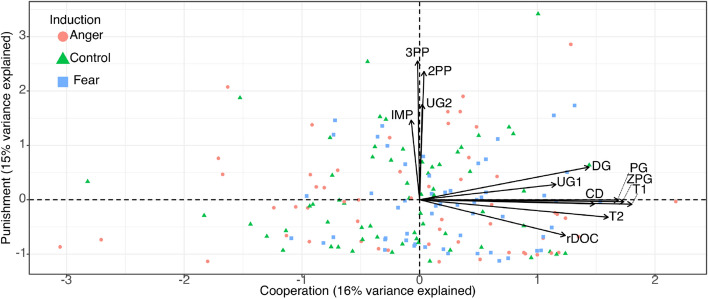
Factor analysis bi-plot. 2 factors, labeled “Cooperation” and a “Punishment”, explain nearly one third (31%) of the variance from 12 different decision environments.

This two factor solution largely corroborated the groupings of economic variables informally suggested by the correlation matrix (Table 1 and Fig. 3): amounts entrusted and returned (i.e., “trustworthiness”), charitable donations, offer sizes in the ultimatum game, transfer size in the dictator game, contributions to public goods, restraint in common resource dilemmas and helping in the Zurich prosocial game, all loaded on the first factor (all loadings > 0.35). Following previous studies^55,56^, we labeled this a “Cooperation factor”. On the other hand, amounts spent to punish others in 2nd and 3rd party punishment games, frequency of rejections in ultimatum and impunity games loaded on the second factor (all loadings > 0.45). Since the top loading variables on this factor were the 2nd and 3rd party punishment games, we called this a “Punishment factor”. Most variables loaded uniquely on the respective factors (mean item complexity = 1.1). Only the rDOC and the DG had above higher complexity (1.5 and 1.3, respectively), loading negatively and positively on the punishment factor, respectively. Böckler et al.^54^ describe two similar factors as an “altruistically motivated prosocial behavior” factor and a “norm-motivated prosocial behavior”, respectively.

**Table 1:** Factor analysis of 12 economic variables: 2-factor solution.

	F1: Cooperation		F2: Punishment		Com	Comp
Trust game (1st mover)	**0.53**		0.00		0.28	1.0
Trust game (2nd mover)	**0.50**		− 0.10		0.26	1.1
Charitable donations	**0.47**		− 0.02		0.22	1.0
Dictator game	**0.45**		0.19		0.24	1.3
Ultimatum game (1st mover)	**0.36**		0.09		0.14	1.1
Public good game	**0.57**		− 0.03		0.32	1.0
Dilemma of the commons	**0.39**		− 0.21		0.19	1.5
Zurich prosocial game	**0.55**		− 0.01		0.30	1.0
2nd party punishment	0.01		**0.74**		0.55	1.0
3rd party punishment	− 0.01		**0.80**		0.64	1.0
Ultimatum game (2nd mover)	0.01		**0.55**		0.30	1.0
Impunity game	− 0.02		**0.46**		0.21	1.0
Proportion variance explained	0.16		0.15			

Standardized loadings (pattern matrix) based upon correlation matrix, communality (“Com”) and complexity (“Comp”) of each variable. Two factors, labeled “Cooperation” and “Punishment”, captured 31% of the total variance.

### The impact of induced fear and anger on latent constructs of cooperation and punishment

**Figure 4:**
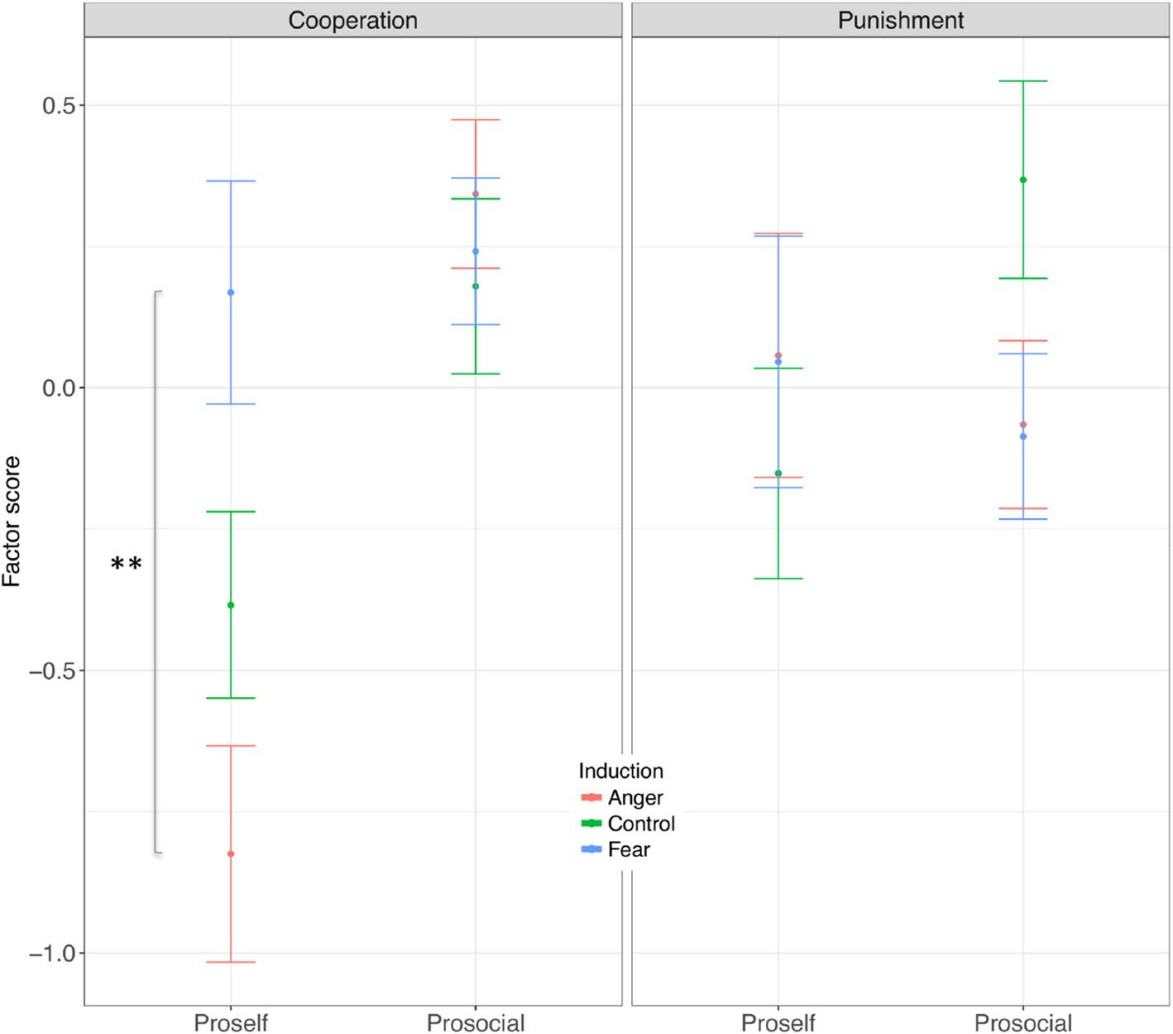
Top: latent measures of cooperation (left) and punishment (right) as a function of induced Fear or Anger, relative to participants in a Control induction. Error bars represent 95th confidence intervals. ***p < 0.001, **p < 0.01, *p < 0.05.

The factor analysis additionally enabled to obtain one pair of scores for each participant. These can also be thought of as a pair of coordinates, determining participants’ position on a “cooperation x punishment space” (Fig. 3). These two scores were submitted to regression analyses. Our primary model of interest (see “Model 1” in “Statistical analyses”) on the cooperation scores revealed a highly significant impact of SVO on cooperation (F_(1,149)_ = 20.222, p < 0.001), a significant main effect of the inductions (F_(2,149)_ = 3.785, p = 0.0.025), and a significant interaction between SVO and the inductions (F_(2,149)_ = 5.482, p = 0.005) (the model estimates are plotted in Fig. 4). Addressing the main effects, contrasts within the model suggested that prosocial participants cooperated more than selfish participants (“Proself–Prosocial”: b = − 0.601, 95% CI [− 0.866 − 0.337], p < 0.001). As for the inductions, cooperation was higher under induced Fear relative to induced Anger (“Anger–Fear”: b = − 0.446, 95% CI [− 0.847 − 0.045], p = 0.024), while neither of the latter differed from Control (“Control–Fear”: b = − 0.307, 95% CI [− 0.703 0.089], p = 0.186; “Anger–Control”: b = − 0.138, 95% CI [− 0.531 0.254], p = 1.00). Finally, contrasts addressing the interaction suggested that the effect of the inductions was driven by proself individuals: while the inductions did not significantly affect levels of cooperation in participants with prosocial orientations (all p_s_ > 0.423, uncorrected), Fear and Anger differentially affected cooperation in participants with selfish orientation. Specifically, in proself individuals, cooperation scores were higher under induced Fear than Anger (“Anger–Fear”: b = − 0.993, 95% CI [− 1.659 − 0.327], p = 0.001), and were non-dissociable between either of the latter and the Control induction (“Anger–Control”: b = − 0.440, 95% CI [− 1.051 0.171], p = 0.250; “Control–Fear”: b = − 0.553, 95% CI [− 1.175 0.070], p = 0.099). In line with this, proself participants cooperated less than prosocial participants in the Control induction condition (“Proself–Prosocial”: b = − 0.564, 95% CI [− 1.010 − 0.117], p = 0.014) and in the Anger induction condition (“Proself–Prosocial”: b = − 1.168, 95% CI [− 1.627 − 0.708], p < 0.001), but not in the Fear induction condition, where proselves cooperated to the same extent as prosocials (“Proself–Prosocial”: b = − 0.073, 95% CI [− 0.540 0.400], p = 0.758). This main finding, namely, that Fear and Anger differentially affect cooperation in selfish individuals, was robust to the inclusion of a number of potential confound variables (including gender, the interaction of gender with the inductions, risk attitudes and happiness and sadness change scores) (see “Model 2” in the Supplementary Material, Supplementary Tables ST4 and ST5); it was also robust to the exclusion of outliers (e.g., by winsorizing the cooperation scores) and to the exclusion of participants that, when explicitly asked in the awareness questionnaire, expressed even irrelevant suspicion about possible connections between the inductions and the economic decisions (see Supplementary Material SM1 for details). Finally, these results held when modeling SVO as a continuous predictor. In particular, a regression model revealed a significant impact of the inductions, of SVO (continuous), and of their interaction in predicting cooperative decisions (all p_s_ < 0.01, see Supplementary Table ST6 in the Supplementary Material). The observed pattern of results was entirely consistent with those illustrated above (see Supplementary Fig. SF1 in the Supplementary Material).

In the same way, we investigated the impact of the inductions, SVO, and their interaction on punishment scores. However here, in contrast to our hypotheses, none of the factors had a significant impact on punishments (SVO: F_(1,149)_ = 0.343, p = 0.559; inductions: F_(2,149)_ = 0.294, p = 0.746; SVO × inductions: F_(2,149)_ = 2.095, p = 0.127). Exploratory contrasts also suggested that the inductions had no significant impact on punishing, neither in selfish nor in prosocial participants (all contrasts p_s_ > 0.142). For comparability with the cooperation model we plot the estimated fixed effects for both social value types in the right panel of Fig. 4. These null-findings were also confirmed by a second linear model incorporating the additional predictors described above (SVO: F_(1,149)_ = 0.124, 0.726; inductions: F_(2,149)_ = 0.531, p = 0.589; SVO × inductions: F_(2,149)_ = 2.162, p = 0.119).

## Discussion

Human cooperative behaviors are modulated by stable inter-individual differences in prosocial or selfish value orientations, yet it remains unclear how these stable values interact with induced or incidental perceptions of threat. To address this, we experimentally induced either anger or fear in individuals with different social value orientations and had them take part in a suite of incentivized decision paradigms that fall along one of two latent factors: cooperative and punishment based decisions^4,27,54–56^. Our results show that induced Fear increases scores on a latent cooperation factor, relative to induced Anger. We also find that this effect is entirely driven by individuals with “selfish” social value orientations. These results extend previous findings in several ways.

In non social decisions, induced fear and anger have been respectively associated with avoidance and approach of risk^18,19,21–26^. Our results extend this by showing that, in the context of social decision making related to cooperation, induced fear is more likely to increase cooperation, relative to induced anger. In addition, given that fear and anger both involve negative valence and high arousal, our findings suggest that the impact of these induced emotions on decision making are unlikely to be entirely explained by mood or arousal alone^19^, nor by “mood reparation”^48^. Rather we suggest that these findings are consistent with motive-based approaches to fear and anger, which link fear and anger to particular motives, such as a defensive or aggressive motives, respectively^15,69^. Under this view, when social contexts are involved, induced fear may lead to social approach oriented behaviors as a protection mechanism, to seek support and acquaintance in the face of danger^38^; while anger can lead subjects to avoid cooperative gestures, as a means to antagonize others^28^.

The fact that this differential effect of induced Fear and Anger is only observed in selfish individuals aligns with the notion that strong cooperative values can buffer the impact of certain motives and emotions^59^: while selfish individuals could behave cooperatively especially when it suits their need for self-protection^44,48^, such a self-serving motive is incoherent with prosocial value orientations. This is also in line with evidence suggesting that fear-induced cooperation is unlikely to stem from genuine care for others. For instance, perceived threats have been shown to increase cooperation with one’s peers or ingroup^70,71^ and stress has been shown to increase cooperation *only* towards closer others^42^. This instrumental perspective of the “tend and befriend” hypothesis^38^ offers a plausible explanation of why this cooperative response to fear is only observed in selfish individuals.

Surprisingly, we do not observe a main effect of induced anger on decisions to punish, which has instead been observed in previous studies^31–34^. With hindsight, we speculate that this can be due to an incorrect selection of a moderating individual difference variable for the punishment domain. In fact, while there was a clear candidate for the value orientation that would predict cooperative behavior, the same was not true for punishing behaviors. Future studies might address whether “social dominance orientation”^72^, or a moderating variable that has been shown to predict punishments in game theoretical paradigms (such as the ‘assertiveness scale’^73^), may be able to better detect the interaction of values and incidental emotions on punishments.

Finally, by taking a factor analytic approach our study is the first to our knowledge to document a link between induced motivational states, social values, and a “domain general” cooperation factor^55^. This approach does not intend to deny important differences between individual decision paradigms, some of which might very well influence how specific decisions are affected by motives and emotions. Rather, we suggest that this approach could provide a relatively unbiased illustration of the effects of induced Fear and Anger on cooperative decision making, by describing how these threat-related emotions affect a source of behavioral variance that is common to many if not all of them^4,54–56^. This factor analytic approach might thus alleviate problems related the reliability and ecological validity of game-theoretical paradigms^53^. Ultimately, it may also contribute to the ongoing integration of economics and psychology^9,74,75^, by helping economists formalize the impact of emotions and motives on economic decision making^76^.

## Conclusions

Plausibly, no psychologist would endorse one of the basic assumptions of neoclassic economic theory: that decisions are driven only by stable context-insensitive preferences^9,77^. However, to integrate psychological and economic frameworks, psychologists should provide empirical evidence as to how important motives and emotions such as fear and anger systematically affect economic decision making. Our results suggest that in individuals with selfish value orientations induced Fear is more likely to increase cooperative behaviors, relative to induced Anger. The finding that selfish individuals drive this differential effect suggests that prosocial values can buffer the impact of certain emotions on cooperation: while selfish individuals may especially increase their cooperative behavior in the face of fear, such instrumental cooperation may conflict with the value orientations of prosocial individuals. Taken together, these findings shed light on the importance of both context-sensitivity and pre-existing differences in prosocial value orientations in determining cooperative behavior. Finally, by capitalizing on a source of variance that is common to many decision contexts and on inter-individual differences, our results highlight how fear-inducing contexts (e.g., cultures or climates of fear) can influence a generalized form of cooperative behavior, and sheds light on which individuals might be more susceptible to this.

## Methods

### Participants

175 participants (82 males, 93 females, mean age = 27.1, SD = 4.8) were assigned either to a “Fear” (N = 56, 25 males, 31 females, mean age = 26.5, SD = 4.4), an “Anger” (N = 56, 26 males, 30 females, mean age = 26.6, SD = 4.1) or a “Control” group (N = 63, 31 males, 32 females, mean age = 27.8, SD = 5.7). A separate group of participants was assigned to two other motive-inductions not relevant to this study, which have been published elsewhere together with the same Control group^56^. Participants were recruited through the Max Planck’s Institute participant database. All studies were advertised, via email, to all eligible participants in the database, namely, participants between the age of 18 and 65, with no history of cognitive, psychiatric or neurological disorder. Participants registered to the studies on a first-come first-served basis. Age and gender were similarly distributed across each group (all pairwise comparisons, p_s_ > 0.1). Participants provided informed consent for the treatment of their anonymized data and all methods were carried out in accordance with relevant guidelines and regulations. Assessments were approved by the Research Ethics Committee (Agreement Number 090-15-09032015) of the University of Leipzig, Germany. Data will be made available upon reasonable request.

### Inductions

Group sessions took place in a computer room with shielded computer cubicles. In all inductions, participants first provided baseline ratings on a number of emotion and motive-related items (see “Induction validation”). Then, they were informed about one of three activities. Participants in the Fear group took part in the anticipatory Trier Social Stress Task (henceforth, “aTSST”)^45,78^, in which they were informed about a simulated job interview requiring them to take part in a series of (stressful) tasks in front of two anonymous interviewers. We chose the anticipatory variant of the TSST because while the standard TSST is known to elicit both fear and anger^79^, the aTSST has been suggested to predominantly enhance anxiety rather than hostility and aggression^78^. To induce Anger, we adapted the “negative feedback” procedure^67,80^ in which participants receive negative (and unfair) feedback on a short personal essay they wrote and anticipated providing feedback to the reviewer’s essay in turn. In the Control condition, participants only anticipated reading a passage of a text. To increase the salience of these activities, participants were accompanied to a different room, one by one. Here, in the Fear induction, they found the two aforementioned interviewers (one male, one female), wearing lab-coats and sitting behind a desk. These interviewers asked participants a set of preliminary questions in a detached fashion (e.g., what job they would like to interview for, and why they thought they would be good candidates for that job). In the Anger induction, participants were shown a single-blind mirror, where they later would have the opportunity to provide feedback to their (unfair) reviewer, via microphone, on his/her essay. In the Control induction, subjects were shown the recording room where they later would be recorded whilst reading the text passage and gave a brief sound check. After returning to the computer room, subjects provided ratings on the same emotion and motive-related items rated previously. A different experimenter then told participants that, while they waited for the activities to be prepared, they would take part in an allegedly different study on economic decision making (see “Game theoretic paradigms”, below). To maintain the inductions salient, half way through the decision making study, participants were asked to take notes for 5 min, to prepare for the activities. After completing the decision making study, participants took part in a written questionnaire probing for awareness of any connection between the two studies (see Supplementary Material SM1, “Experimental demand questionnaire”). Finally, participants were fully debriefed and paid for their participation and for one of their decisions. The whole experimental session was self-paced and lasted 1 h and 30 min on average.

### Induction validation

Before and after being introduced to the induction-specific activities (see “Inductions”), participants provided baseline (“pre-induction”) and test (“post-induction”) ratings indicating how well a list of fear and anger-related words described their motivation or feelings (with visual analogue scales ranging from − 350 to 350). The fear items were (here translated from German) “apprehensive”, “afraid”, “timid”, “nervous”, “panic-stricken”, “overcautious”, “frightened”, “reserved”, while the anger items were “aggressive”, “angry”, “offended”, “irritable”, “argumentative”, “tempestuous”, “spirited”. These items were chosen because they best-discriminate the target constructs from related but distinct constructs^81^. Previous work using emotional inductions prevalently controlled for constructs of interest only, yet it appears plausible that, an Anger induction may concurrently increase feelings of power in some individuals, or that a Fear induction may also increase feelings of achievement, given that it involves a simulated job interview. To address this, in addition to the emotions of interest (i.e., fear and anger), the questionnaire also probed five motive-related measures: achievement, affiliation, care, power and consumption; and two affect-related measures: happiness (i.e., positive affect) and sadness (negative affect) (see Supplementary Material SM2 for the list of all items). Overall, the questionnaire consisted of 63 items composing 9 measures (7-motive related and 2 affect related constructs). The order of all items was fully randomized for each participant, who rated 7 items per page. To analyze this data, we first subtracted the pre-induction ratings of each item from the corresponding post-induction ratings and then averaged over items pertaining to the same construct, thus yielding 9 “change scores”. Finally, to validate the inductions, we compared these difference scores between the inductions (see “Statistical analyses”).

### Game theoretic paradigms

To measure cooperation and punishment we had subjects take part in a suite of paradigms that have been found to factor together in previous studies^54–56^. Specifically, as candidate contributors to a “cooperation factor”, we had participants take part in a dictator game (in which they decided how much money, if any, to transfer to a passive recipient), a trust game (in which, as 1st movers, participants decided how much to entrust to a second mover, and as 2nd movers decided how much to return to the 1st movers), a charitable donations game (in which participants decided how much to donate to various charities), a public good game (in which they decided how much money to contribute to a public good), a common resource dilemma (in which they decided how much to take from a common resource) and the “Zurich prosocial game” (in which participants decided how much to help their counterparts in a virtual maze). As for the punishment-related games, we adopted the 2nd and 3rd party punishment game (in which participants observed how much another player transferred to themselves or a third party—2nd and 3rd mover variants, respectively—and, on the basis of this, decided how much money, if any, to invest to “punish” them, that is, to decrease their payoff), an ultimatum game (in which, as 1st movers, participants made a proposal on how to split a monetary prize to a responder, knowing that responders would then have two options: if they accepted the monetary prize was split as proposed, if they rejected, both players received nothing), and an impunity game (which is identical to the ultimatum game with the exception that, if second movers rejected an offer, first movers still retain what they proposed to keep for themselves). Following our previous study^56^, we first obtained one measure for each paradigm (averaging over measures in the case of multiple trials). We then subjected the resulting 12 measures to factor analysis (see “Statistical analyses”). Finally, to control for the potential impact of our inductions on social decisions net of any effect they may have on non-social decisions, we measured risk attitudes by means of a lottery task. This task was taken from Dawans and colleagues^39,45^, and involved a series of binary decisions in which participants chose between two lotteries with similar expected value but different levels of risk. The frequency of choices of the riskier lottery was used as a measure of participants’ non-social risk attitudes. All economic games were divided in two blocks to avoid potential spill-over effects between superficially similar games (such as 2nd and 3rd party punishment, see Supplementary Material SM3 for full details on block composition). The order of games within a block was fully randomized and the order of blocks was counterbalanced between participants. Participants were informed that they would be paid for one randomly determined decision, at the end of the experiment. Full details on each of the decision paradigms are available in the Supplementary Material (Supplementary Tables ST1 and ST2). Instructions are available upon request.

### Social value orientation

To measure inter-individual differences in social values we used the social value orientation task (“SVO”)^2,3^. In order to reduce the possibility that the inductions could affect the SVO scores, these were measured on a different day. Specifically, two weeks after the participants came to the lab to take part in the economic decisions, they were sent an email linking them to the SVO questionnaire (to be done online). We used the SVO “slider measure”, as this has been suggested to be more reliable than previous measures^82^ and we focused on the 6 primary items because the secondary items are mostly required to distinguish between finer types of prosocial orientation, which were outside of the scope of this study. Each of these six primary items require subjects to choose between nine different point allocations to themselves and anonymous others (e.g., between option A: {100 for self and 50 for other} vs. option B: {85 for self, 85 for others}, etc.). Following previous research^83^, we divided participants into “proself”, also called “selfish” participants (a classification which combines subjects displaying individualists and competitive values), and “prosocial” participants (combining participants with prosocial and altruistic values). These aggregations are typically made because of the relatively low numbers of altruists and competitors that are observed^5^. All participants had accepted to take part in this additional online component of the experiment but 20 participants (three in the Anger induction, three in the Fear induction, 14 in the Control induction) never got back to our invitation emails. Consequently, the SVO scores of these participants, but not their economic decisions, are missing. The SVO scores did not differ between any of the groups (all p_s_ > 0.17). In the Anger, Control, and Fear conditions, respectively, we identified n = 17, 23 and 16 proself participants and n = 36, 26 and 37 prosocial participants. Finally, our results also held when modelling SVO as a continuous predictor (Supplementary Table ST6and Supplementary Fig. SF1)^84^.

### Statistical analysis

For induction validation, we used a mixed effect model predicting change scores in self reported ratings based on the induction (with levels: “Fear”, “Anger”, “Control”) the motivational/affective state (9 levels) and their interaction. These factors were modeled as fixed effects, while participant IDs were used as random intercept terms, to account for the fact that observations were clustered at the subject level. Planned contrasts within this model focused on the emotions of interest, namely, whether changes in fear were higher in the Fear induction relative to the other inductions, and whether changes in anger differed between the Anger induction and the other inductions. Additional exploratory contrasts investigated the remaining 7 change scores.

To investigate our main question of interest (i.e., whether induced Fear and Anger differentially affect latent factors of social decision making), we first aimed to obtain a reliable factor scores for the 12 decision environments, following the methods used in our previous work^56^. More specifically, we first investigated the optimal amount of factors to retain using a number of standard indices^68^, and then tested whether the resulting factor structure was stable across the inductions^85^. Factor analysis was performed with the “fa” function (in the “Psych” package)^86^, while the stability of the factor structure was performed with the “measurementInvariance” function (in the “semTools” package)^85^. Finally, we extracted participants’ scores on each of the obtained factors, and investigated whether these differed between the inductions, using multiple regression models. We tested two models on each factor score. “Model 1” addresses our main hypotheses of interest, thus predicting participants’ factor scores on the basis of the induction (three levels: Fear, Anger and Control), participants’ SVO category (two levels: prosocial and proself), and their interaction. “Model 2” aimed to assess robustness of the results by additionally controlling for a number of potential confound variables, including gender—which has elsewhere been shown to have a potential impact on decisions to cooperate or punish^71,87^—as well as the (standardized) measure of subjects’ risk attitudes as measured by the lottery task described above. We report the results of Model 1 in the results section and the results of Model 2 in the Supplementary Material. For all models, we report the results of the omnibus tests (as assessed by type III Anova) and further qualify these by means of contrasts, of which we report 95% confidence intervals p-values (as computed in the “lsmeans” package^88^). P-values are Bonferroni corrected, unless otherwise noted. All analyses were carried out in R^89^. Stimuli were prepared and administered in Presentation (Neurobehavioral Systems, Inc.).

## Supplementary Information

Supplementary Information.
