# Supplementary material for: Cooperation across multiple game theoretical paradigms is increased by fear more than anger in selfish individuals
Source: Sci Rep. 2021 Apr 30;11:9351. doi: 10.1038/s41598-021-88663-0 (PMC8087760; doi:10.1038/s41598-021-88663-0)
Supplement: Supplementary file 1 — Supplementary Information. [file 41598_2021_88663_MOESM1_ESM.docx]

**Supplementary Material**

**Cooperation across multiple game theoretical paradigms is increased by fear more than anger in selfish individuals**

Chierchia G.^a*^, Parianen Lesemann F. H.^b^, Snower D.^c,d^ Singer T. ^e^

^a.^ University of Cambridge, Cambridge, United Kingdom

^b.^ Utrecht University, Utrecht, Netherlands;

^c.^ Department of Economics, Hertie School of Governance, Berlin, Germany;

^d.^ Blavatnik School of Government, Oxford, United Kingdom;

^e.^ Social Neuroscience Lab, Max Planck Society, Berlin, Germany

* Corresponding author

***SM1. Experimental demand questionnaire***

To control whether our results might have been affected by experimental demand, at the end of the experimental session, participants took part in a questionnaire probing their possible insights into the objectives of the experiment.

*Material.* The questionnaire was inspired from Bargh & Chartrand's (2000) “funneled” debrief questionnaire^1^, in which participants are gradually made aware of elements in the experimental environment that might have affected their behavior. Specifically, participants provided open answers to the following 7 questions:

*Methods.* Two experimenters independently scored the answers and, for each of the questions above, provided a rating, from 1 (“no sign of suspicion”) to 4 (“relevant understanding”), indicating how much they believed the participant had insight into the objective of the induction. After testing for inter-rater reliability and averaging over their ratings, we then re-ran our main analysis of interest using two exclusion criteria: the first excluded participants that understood how the announced activities were intended to affect their economic decisions (e.g., “You wanted to test how stress affects decision making”) (a score of 4), while a second more stringent criteria excluded participants that admitted that the activities could have affected their decisions, but not in a way that was pertinent to our experimental hypotheses (e.g., “You wanted to see how we deal with distraction”) (a score of 3). If the participants provided no answer on a given question, or an irrelevant answer, raters were instructed to provide no rating.

1. What is your next task?

2. What do you think are the goals this study?

3. Do you think one part of the study might have influenced another? If so, how and why?

4. Did you notice anything unusual about the experiment?

5. At any moment did you have the feeling of being deceived?

After the 5^th^ question, participants were told that anticipated activities would actually not take place.

6. Did you already doubt that you would not [see the puppies/take part in the business simulation?]

7. Now that you know, what do you think the goal of this study was?

This scoring system was used for all the questions above, except for question 1 (“what’s the next task?”). For this question, a score of 1 indicated that the participant answered as expected (e.g., “going to take part in the interview” or “going to give feedback to my reviewer”), while a score of 2 indicated that participants expressed doubts that the activity would actually take place. However, neither of the raters ever provided a score of 2 for this question, which we thus disregarded for the remaining analysis. Consequently, we focused on questions 2 through 5, that is, on any question that was asked before revealing that the announced activities would actually not take place. Since these items had high inter-rater reliability (Pearson’s correlations between r=0.6 and 0.95, all p_s_<0.001), we averaged between the ratings provided by the two raters.

*Results.* Ratings suggested that five participants in the Fear induction and five participants in the Anger induction expressed relevant understanding on how the announced activities were intended to affect their behavior. We thus re-ran the main analyses of interest after excluding these participants and results were unaffected. Specifically, the cooperation model described in the paper continued to suggest that, in selfish participants only, induced Fear resulted in higher cooperation scores than induced Anger (b= -0.744, 95% CI [-1.438 -0.051], p<0.05), while neither of the latter differed from Control (both p_s_>0.13). Even when excluding participants that provided irrelevant explanations as to how the activities might have affected their decisions (which, together with the previously excluded ones amounted to N=23 in the Anger induction and N=10 in the Fear induction), induced Fear significantly increased cooperation scores, relative to induced Anger, in selfish participants (b= -0.889, 95% CI [-1.714 -0.064], p<0.05).

***SM2. Induction validation items***

Items contributing to the fear and anger-related measures of interest are listed in the paper. Below we provide items forming the other 7 control measures, 5 of which measure motives and 2 affect. The items for the five motives were the following: care (“caring”, “protective”, “kind-hearted”, “cordial”, “helpful”, “affectionate”, “sympathetic”, and “consoling”), power (“mighty”, “dominant”, “authoritarian”, “firm”, “influential”, “condescending” and “officious”), achievement (“hard-working”, “industrious”, “capable”, “efficient”, “ambitious”, “success-driven”, “obstinate”, “productive”), affiliation (“entertaining”, “ingratiating”, “excluded”, “conventional”, “attached”, “obliging”, “popular”) and consumption (“consumerist”, “hoarding”, “avaricious”, “greedy”, “materialistic”, “cheap”, “pleasure-seeking”, “acquisitive”, “desire to buy”, “gluttonous”). In addition to these motive-related constructs, positive and negative affect was measured by asking participants to provide ratings on a number of items related either to happiness (“content”, “happy”, “overjoyed”, “pleasant”, “enthusiastic”) or sadness (“sad”, “downcast”).

***Supplementary Table ST1: Game theoretic paradigms***

For each game, we indicate in parenthesis which variable was derived: DG (amount transferred), CD (average donation size), T1 (amount entrusted), T2 (average amount returned), UG1 (amount proposed), UG2/Imp (percentage of rejections), rDOC (average amount of taking, after reverse scaling, in order to favor comparability with the public good game), PG (average contribution), ZPG (percentage of decisions to help). For each decision, participants were told that they would be randomly re-matched to another participant in the room. “*” indicate games that we piloted here but were unrelated to this study. ** indicate games that were analyzed for consistency with our earlier study, which suggested they either did not load on any factor (“SH”) or rendered the factor solution unstable between the inductions (“MBG” and “EG”).

|  | **Game theoretical paradigms** | **Description** | **Parameters**  **(n=number of trials)** |
| --- | --- | --- | --- |
| 1 | Dictator game (“DG”)^2^ | Subjects are endowed with a euro amount and decide how much, if any, of their endowment to transfer to an anonymous counterpart. | Endowment=€10.00  (n=1) |
| 2** | Stag hunt (“SH”)^3^ | Randomly paired participants are to choose between (the same) two options: a safe but low paying option and a potentially higher paying but uncertain option. If both choose the high paying option, both earn the high payoff. However, if one chooses the high paying option and the other does not, the one that chose the high paying option receives nothing at all, while the low paying option results in a secure payoff (i.e., regardless the choice of one’s counterpart). Participants made several decisions but never received feedback on their outcome. The value of the high paying option was kept fixed, while the value of the low paying option varied for each decision. | Low paying option value varied between 0 and 15€, in steps of 1€  High paying option value was kept fixed and was always worth 15.00€ o 0, depending on the choice of one’s counterpart.  (n=16, randomized order) |
| 3 | Public good game^4^ | Groups of three participants decide how much of an endowment to contribute, if anything, to a “pubic good”. Contributions are summed and multiplied by a given factor and the resulting amount is split equally among players, regardless of how much they contributed. | Endowment=€30.00  Multiplier factor = {2,3}  (n=2, no randomization) |
| 4 | Ultimatum game (as 1^st^ mover, “UG1”, and 2^nd^ mover, “UG2”)^5^ | As first movers (the “proposer”), participants decide how much of an endowment, if anything, to propose to a second mover. | Endowment=€10.00  (n=1) |
| 5 |  | As second movers (“recipients”), participants observe the proposal and decide whether to “accept” or “reject”. If accepted, each player receives the endowment share proposed by the proposer. If rejected, both players earn nothing. | Observed proposals = {0.2, 1.1, 1.7, 3.1, 4.1, 4.9}  (n=6, randomized order) |
| 6 | Third party punishment (“3PP”)^6^ | A first mover decides how much of an endowment to transfer to a randomly matched counterpart.  Participants play the role of a 3^rd^ unaffected party, who observe how much is transferred and decide how much of an endowment to spend, if anything, to punish the 1^st^ mover.  This amount is multiplied a given factor and subtracted from the 1^st^ mover’s payoff. Participants only played the 2PP as the 3^rd^ unaffected party. | 1^st^ mover endowment=€15.00  2^nd^ mover endowment=€5.00  Observed transfers = {0.2, 1.6, 2.5, 3.6, 6.1, 7.4}  Multiplier factor=3  (n=6, randomized order) |
| 7 | Trust game (as 1^st^ movers, “TG1” and 2^nd^ movers, “TG2”)^7^ | A 1^st^ mover (“truster”) decides how much of an endowment, if anything, to transfer to a 2^nd^ mover (“trustee”). This amount is multiplied by a given factor and transferred to the 2^nd^ movers. | Endowment=€10.00  Mulitpier factor=3  (n=1) |
| 8 |  | A 2^nd^ mover (“trustee”) observes how much was transferred and decides how much of the transfer, if anything, to transfer back to the 2^nd^ mover. | Observed transfers={1.6, 3.3, 5.1, 6.7, 8.3, 9.9}  (n=6, randomized order) |
| 9** | Money burning game (“MBG”)^8^ | A pair of randomly matched counterparts decide how much of an endowment to spend to decrease the payoff of their counterparts. This amount is multiplied by a given factor and subtracted from the counterpart’s payoff. Subjects earn nothing from “burning” their counterparts’ payoff. | Endowment=€10.00  Multiplier factor={2,3,4,5}  (order randomized)  (n=4, randomized order) |
| 10** | Entry game (“EG”)^9^ | Randomly paired participants are to choose between the same two options: a safe but low paying option and a potentially higher paying but uncertain option. If both players choose the higher paying option, both earn 0. The only way for a player to earn the high paying option is if he/she chooses it and the counterpart does not. On the other hand, the low paying option results in a secure payoff, regardless the choice of one’s counterpart. Participants made several decisions but never received feedback on the outcome of any of them. The value of the high paying option was kept fixed, while the value of the low paying option varied for each decision. | Low paying option value varied between 0 and 15€, in steps of 1€  High paying option value was kept fixed at 15.00.  (n=16, randomized order) |
| 11 | Dilemma of the commons (“DOC”)^10^ | Groups of three participants decide how much of take from a common resource. The amounts taken are then summed. If this sum exceeds a certain threshold, all players receive nothing. If the threshold is not exceeded, each player keeps what they took and the remaining sum in the common resource is first multiplied by a given factor and then split equally among the players, independently of what they took. | Common resource value=€30.00  Threshold=€18.00  Multiplier factor={2,3}  (n=2, no randomization) |
| 12 | Second party punishment (“2PP”)^11^ | A first mover decides how much of an endowment to transfer to a randomly matched counterpart.  Participants play the role of the 2^nd^ mover unaffected party, who observe how much is transferred and decide how much of an endowment to spend, if anything, to punish the 1^st^ mover.  This amount is multiplied a given factor and subtracted from the 1^st^ mover’s payoff. Participants only played the 2PP as second movers. | Endowment=€5.00  Multiplier factor=3  Observed transfers = {0.3, 1.5, 2.6, 3.5, 6.1, 7.5}  (n=6, randomized order) |
| 13 | Impunity game (“Imp”)^12,13^ | The impunity game is identical to the ultimatum game with one exception: if recipients reject an offer, they earn nothing (as in the ultimatum game) however the proposer still keeps what he/she proposed. Subjects only played the impunity game as second movers. | Observed proposals={0.1, 0.9, 1.6, 2.9, 3.9 4.8}  (n=6, randomized order) |
| 14 | Charitable donations (“CD”)^14,15^ | Subject read short descriptions of several real life charitable organizations and decided how much of an endowment to donate. They were informed that a random decision would be selected and that they could keep 20% of what they did not donate. | Endowment=€50.00  (n=13, randomized order) |
| 15 | Zurich prosocial game (“ZPG”)^14,16^ | Two participants simultaneously navigate a computer maze with the objective of reaching a treasure worth a monetary prize. There are treasures, one for each participant, and there is no way for a player to obtain both. Occasionally, paths are blocked by locked doors and participants are to decide whether to you use one of their limited keys to open the door for their co-players or not. | Prize=€0.5  (n=8, randomized order) |
| 16* | Asymmetric dictator game (“ADG”) (adapted from^17^) | The asymmetric dictator game is identical to the dictator game (see 1 in this table) with the exception that subjects (i.e., the “dictators”) are told that recipients will never now any game ever took place. Instead, recipients are told that any additional money they receive is the result of a lottery. | Endowment=€10.00  (n=1) |
| 17* | Compliance (“Comp”)  (adapted from^18^) | Identical to the charitable donations (see 14 in this table) with the exception that participants are informed about the average donations of other participants, before making their choices. | Endowment=€50.00  (n=13, randomized order) |

***Supplementary Table ST2:* non-social economic paradigms.** For each paradigm, we indicate, in parenthesis, which variable was derived: risk (the percentage of risky choices), loss-aversion (the percentage of choices to gamble), temporal discounting (the percentage of patient decisions). * Indicate two tasks not relevant to the hypotheses of this study.

|  | **Game** | **Description** | **Game parameters**  **(number of trials)** |
| --- | --- | --- | --- |
| 1 | Risk^19^ | Participants choose between two options, specifically two mixed gambles (“A” or “B”), each offering a 50-50 chance to gain/lose different monetary amounts. The two options were similar in expected value but differed in their “riskiness”/variance. | The gamble parameters were adapted from von Dawans et al. (2012) and were the following:   \| A \| B \| \| --- \| --- \| \| +5.2/+0.4, \| +2.7/+2.3 \| \| +6.2/+0.4 \| +2.7/+2.3 \| \| +3.7/-1.1 \| +1.2/+0.8 \| \| +5.7/-1.1 \| +1.2/+0.8 \| \| +4.2/-0.4 \| +2.7/+2.3 \| \| +7.2/-0.4 \| +2.7/+2.3 \| \| +4.7/-1.1 \| +1.2/+0.8 \| \| +6.7/-1.1 \| +1.2/+0.8 \|   (n=8, randomized order) |
| 2* | Loss aversion^20^ | Participants are required to make a series of binary decisions, on whether to accept or reject a gamble. If participants accept the gamble they can either win or lose a given monetary amount, with equal probability. | The parameters were adapted from Gächter et al. (2007).  The gain magnitude was always kept fixed at €6.00.  Loss magnitude={-1,-2,-3,-4,-5,-6,-7}  (n=7, randomized order) |
| 3* | Temporal discounting^22^ | Participants are to choose between a sooner but lower paying option and later but higher paying option. | Parameters were adapted from Rustichini et al. (2016).  The later-paying option value was kept fixed at 80€.  The sooner paying option value varied between 45€ and 75€, in steps of 5€.  There were 4 possible moments in which the sooner and later rewards could be delivered, these are listed in the table below:   \| Sooner \| Later \| \| --- \| --- \| \| now \| tomorrow \| \| now \| in 1 week \| \| in 1 week \| in 1 week and 1 day \| \| in 1 week \| in 2 weeks \|   (n=28, randomized order) |

***SM3. Pseudo-randomization of the economic games. Adapted, with permission from Chierchia and colleagues***^23^***.***

The economic games were distributed over two blocks and their order was pseudo-randomized. Specifically, there were three pairs of games that we believed were more likely to influence one another, as they were superficially similar to one another: 1) the 2nd player and 3rd party punishment games, 2) the stag hunt and the entry game and 3) the dictator game and the asymmetric dictator game. In order to minimize potential spill-over between these superficially similar games, games within each of these pairs were always placed in distinct blocks (thus temporally farther away from one another). In addition to this, the charitable donations game was always placed at the end of the second block, as it adopted potentially compassion evoking charity descriptions that we suspected could affect subsequent choices. Finally, the Zurich Prosocial game was played after all of the economic games, as this was designed to investigate a more ecological form of decision making^16^. The order of all the other economic games was fully randomized over the 2 blocks, for each participant, and the order of blocks was counter-balanced across participants.

**Supplementary Table ST3. Contrasts between change scores of non-interest.** Significant differences between the Fear and Anger inductions are in bold.

| Contrast | Item | Estimate | Lower CI | Upper CI | P-value | Bonferroni-adj. |
| --- | --- | --- | --- | --- | --- | --- |
| anger - control | achievement | -33.046 | -64.901 | -1.191 | 0.042 | 0.966 |
| anger - fear | achievement | -1.783 | -34.562 | 30.995 | 0.915 | 1 |
| control - fear | achievement | 31.263 | -0.592 | 63.117 | 0.054 | 1 |
| anger - control | affiliation | -26.018 | -57.873 | 5.836 | 0.109 | 1 |
| anger - fear | affiliation | -21.809 | -54.587 | 10.969 | 0.192 | 1 |
| control - fear | affiliation | 4.21 | -27.645 | 36.064 | 0.795 | 1 |
| control - fear | anger | -27.646 | -59.501 | 4.208 | 0.089 | 1 |
| anger - control | care | -36.645 | -68.5 | -4.791 | 0.024 | 0.552 |
| anger - fear | care | -19.547 | -52.325 | 13.231 | 0.242 | 1 |
| control - fear | care | 17.098 | -14.756 | 48.953 | 0.293 | 1 |
| anger - control | consumption | -20.146 | -52 | 11.709 | 0.215 | 1 |
| anger - fear | consumption | -17.252 | -50.03 | 15.526 | 0.302 | 1 |
| control - fear | consumption | 2.894 | -28.961 | 34.749 | 0.859 | 1 |
| anger - control | fear | 49.756 | 17.901 | 81.611 | 0.002 | 0.046 |
| anger - control | happiness | -135.64 | -167.495 | -103.785 | <0.001 | <0.001 |
| anger - fear | happiness | -53.007 | -85.785 | -20.229 | 0.002 | 0.046 |
| control - fear | happiness | 82.633 | 50.778 | 114.488 | <0.001 | <0.001 |
| anger - control | power | 46.58 | 14.726 | 78.435 | 0.004 | 0.092 |
| anger - fear | power | 17.776 | -15.003 | 50.554 | 0.288 | 1 |
| control - fear | power | -28.805 | -60.66 | 3.05 | 0.076 | 1 |
| anger - control | sadness | 93.075 | 61.221 | 124.93 | <0.001 | <0.001 |
| anger - fear | sadness | 74.205 | 41.427 | 106.984 | <0.001 | <0.001 |
| control - fear | sadness | -18.87 | -50.725 | 12.985 | 0.245 | 1 |

**Supplementary Table ST4. Model 2 results.** Linear multiple regression modeling cooperation scores based on the factors of interest and a number of potential confound variables. Significant effects are in bold.

| **Variable** | **Df1** | **Df2** | **F-value** | **P-value** |
| --- | --- | --- | --- | --- |
| Intercept | 1 | 143 | 0.203 | 0.653 |
| Induction | 2 | 143 | 3.355 | 0.038 |
| **SVO** | **1** | **143** | **18.241** | **<0.001** |
| Sex | 1 | 143 | 2.799 | 0.097 |
| Happiness change | 1 | 143 | 0.761 | 0.385 |
| Sadness change | 1 | 143 | 0.837 | 0.362 |
| Risk attitudes | 1 | 143 | 2.484 | 0.117 |
| **Induction X SVO** | **2** | **143** | **3.816** | **0.024** |
| Induction X Sex | 2 | 143 | 1.871 | 0.158 |

Supplementary **Table ST5. Contrasts within model 2.** Significant effects are in bold.

| **Contrast** | **SVO** | **Estimate** | **Lower CI** | **Upper CI** | **P-value** | **Bonferroni adjusted** |
| --- | --- | --- | --- | --- | --- | --- |
| Anger - Control | Proself | -0.261 | -0.78 | 0.257 | 0.321 | 1 |
| **Anger - Fear** | **Proself** | **-0.819** | **-1.371** | **-0.267** | **0.004** | **0.024** |
| Control - Fear | Proself | -0.558 | -1.07 | -0.046 | 0.033 | 0.198 |
| Anger - Control | Prosocial | 0.299 | -0.152 | 0.749 | 0.192 | 1 |
| Anger - Fear | Prosocial | 0.095 | -0.287 | 0.478 | 0.623 | 1 |
| Control - Fear | Prosocial | -0.203 | -0.626 | 0.219 | 0.343 | 1 |

**Supplementary Table ST6. Social value orientation as a continuous predictor.** Multiple regression model assessing the impact of social value orientation (“SVO”), the inductions and their interaction on cooperation scores.

| **Variable** | **Df1** | **Df2** | **F-value** | **P-value** |
| --- | --- | --- | --- | --- |
| Intercept | 1 | 149 | 28.302 | <0.001 |
| Induction | 2 | 149 | 7.704 | 0.001 |
| SVO | 1 | 149 | 35.069 | <0.001 |
| Induction X SVO | 2 | 149 | 6.709 | 0.002 |


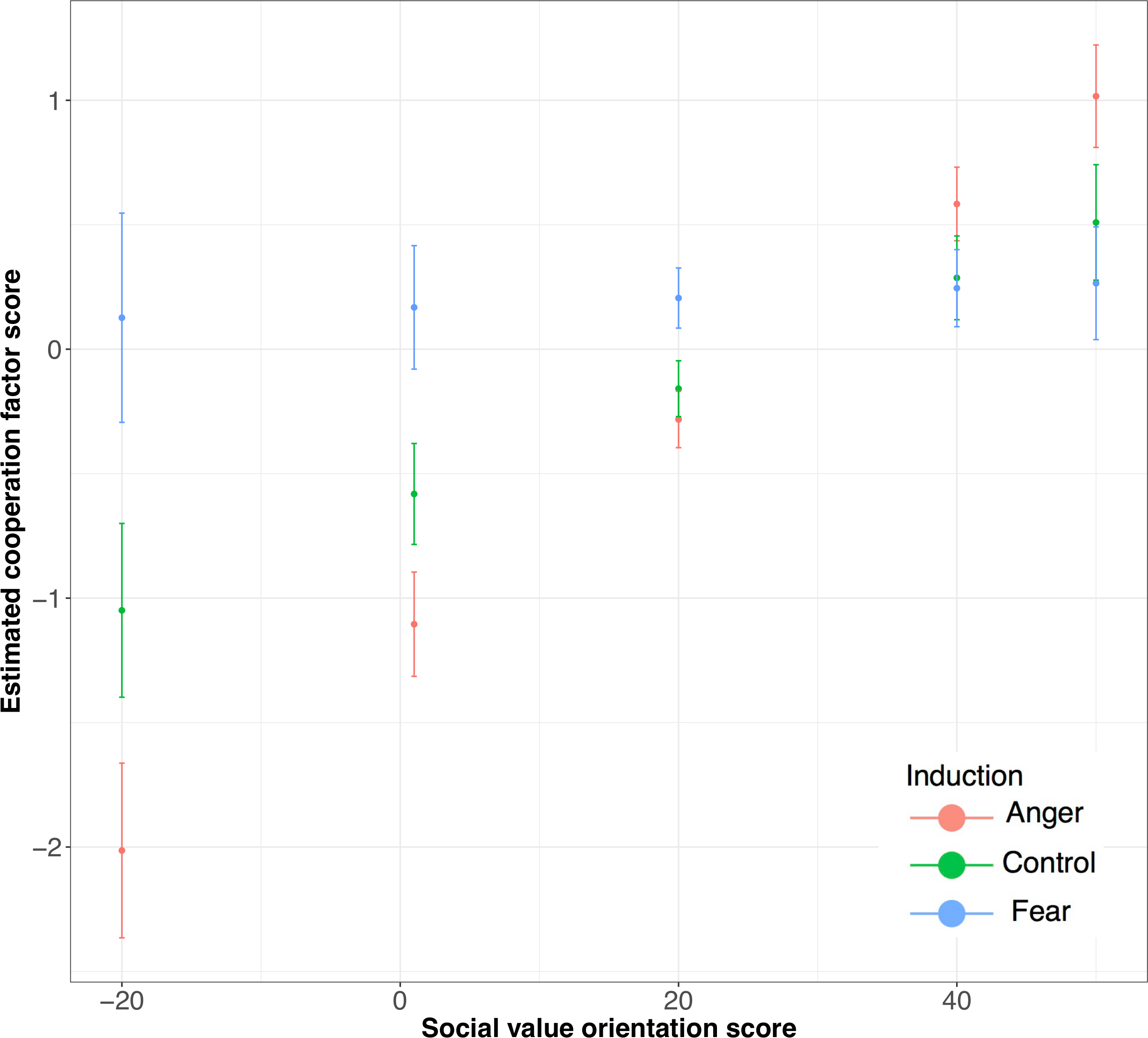


***Supplementary Figure SF1. Social value orientation as a continuous predictor.*** Estimated cooperation scores based on motivational inductions and their interaction with the social value orientation score.

***References***

1. Bargh, J. & Chartrand, T. The mind in the middle. in *Handbook of research methods in social and personality psychology* 253–285 (2000).

2. Engel, C. Dictator games: A meta study. *Exp. Econ.* (2011).

3. Devetag, G. & Ortmann, A. When and Why ? A Critical Survey on Coordination Failure in the Laboratory 1. 1–21 (2006).

4. Zelmer, J. Linear public goods experiments: A meta-analysis. *Exp. Econ.* (2003).

5. Larney, A., Rotella, A. & Barclay, P. Stake size effects in ultimatum game and dictator game offers: A meta-analysis. *Organ. Behav. Hum. Decis. Process.* **151**, 61–72 (2019).

6. Fehr, E. & Fischbacher, U. Third-party punishment and social norms. *Evol. Hum. Behav.* **25**, 63–87 (2004).

7. Johnson, N. & Mislin, A. Trust games: A meta-analysis. *J. Econ. Psychol.* (2011).

8. Zizzo, D. J. & Oswald, A. J. Are people willing to pay to reduce others’ incomes? *Ann. Econ. Stat.* 39–65 (2001).

9. Camerer, C. *Behavioral game theory: Experiments in strategic interaction*. (Princeton University Press, 2003).

10. Ostrom, E., Dietz, T., Dolsak, N., Stern, P. & Stonich, S. *The drama of the commons*. (2002).

11. Fehr, E. & Gächter, S. Fairness and retaliation: The economics of reciprocity. *J. Econ. Perspect.* (2000).

12. Bolton, G. & Zwick, R. Anonymity versus punishment in ultimatum bargaining. *Games Econ. Behav.* (1995).

13. Yamagishi, T. *et al.* The private rejection of unfair offers and emotional commitment. *Proc. Natl. Acad. Sci. U. S. A.* **106**, 11520–11523 (2009).

14. Böckler, A., Tusche, A. & Singer, T. The Structure of Human Prosociality Differentiating Altruistically Motivated, Norm Motivated, Strategically Motivated, and Self-Reported Prosocial Behavior. *Soc. Psychol. Personal. Sci.* **7**, 530–541 (2016).

15. Hare, T. a, Camerer, C. F., Knoepfle, D. T. & Rangel, A. Value computations in ventral medial prefrontal cortex during charitable decision making incorporate input from regions involved in social cognition. *J. Neurosci.* **30**, 583–90 (2010).

16. Leiberg, S., Klimecki, O. & Singer, T. Short-term compassion training increases prosocial behavior in a newly developed prosocial game. *PLoS One* **6**, e17798 (2011).

17. Dana, J., Cain, D. M. & Dawes, R. M. What you don’t know won’t hurt me: Costly (but quiet) exit in dictator games. *Organ. Behav. Hum. Decis. Process.* **100**, 193–201 (2006).

18. Klucharev, V., Hytönen, K. & Rijpkema, M. Reinforcement learning signal predicts social conformity. *Neuron* **61**, 140–151 (2009).

19. Bohnet, I. & Zeckhauser, R. Trust, risk and betrayal. *J. Econ. Behav. Organ.* (2004).

20. McCusker, C. & Carnevale, P. Framing in resource dilemmas: Loss aversion and the moderating effects of sanctions. *Organ. Behav. Hum. Decis.* (1995).

21. Gächter, S., Johnson, E. & Herrmann, A. Individual-level loss aversion in riskless and risky choices. (2007).

22. Stevens, J. & Hauser, M. Why be nice? Psychological constraints on the evolution of cooperation. *Trends Cogn. Sci.* (2004).

23. Chierchia, G., Lesemann, F. H. P., Snower, D., Vogel, M. & Singer, T. Caring Cooperators and Powerful Punishers: Differential Effects of Induced Care and Power Motivation on Different Types of Economic Decision Making. *Sci. Rep.* **7**, 11068 (2017).
